# Illness acceptance and medication adherence in patients with diabetes in Poland: a cross-sectional study

**DOI:** 10.3389/fpubh.2025.1690434

**Published:** 2025-11-20

**Authors:** Agnieszka Pluta, Alicja Marzec, Marzena Humańska, Edyta Kobus, Mariola Głowacka

**Affiliations:** 1 Faculty of Health Sciences, Ludwik Rydygier Collegium Medicum in Bydgoszcz of the Nicolaus Copernicus University in Toruń, Bydgoszcz, Poland; 2 Tadeusz Borowicz Provincial Infectious Diseases Hospital in Bydgoszcz, Bydgoszcz, Poland; 3 Collegium Medicum, The Mazovian University in Płock, Płock, Poland

**Keywords:** illness acceptance, medication adherence, health psychology, type 2 diabetes, self-management, illness perception, psychological adaptation

## Abstract

**Background:**

Diabetes management strongly depends on patient adherence to treatment, which is influenced by psychological factors such as illness acceptance.

**Objective:**

This study aimed to assess the levels of illness acceptance and medication adherence among patients with diabetes and to examine the relationship between these factors.

**Methods:**

A cross-sectional study was conducted among 190 adult patients with type 1 or type 2 diabetes attending outpatient clinics in Poland. Patients were approached and invited to participate in the study when they attended their scheduled outpatient clinic appointments. A total of 256 patients were invited, of whom 36 declined. Out of the 220 questionnaires returned, 190 were fully completed and included in the final statistical analysis. The study was conducted between May 16, 2017, and the end of 2017. Illness acceptance was measured using the Acceptance of Illness Scale (AIS), and adherence was assessed with the Morisky Medication Adherence Scale (MMAS-8). Sociodemographic and clinical data were also collected.

**Results:**

The mean illness acceptance score was 29.16 ± 6.53, with 53.2% of participants demonstrating a high level of acceptance. Medication adherence was moderate according to the MMAS-8 classification (6.49 ± 1.5 points). Illness acceptance was positively correlated with adherence (*R* = 0.29, *p* < 0.001). Patients who reported a preference for structured education and psychological support showed higher adherence scores, emphasizing the importance of patient-centered interventions.

**Conclusion:**

The findings indicate that higher illness acceptance is associated with better adherence in patients with diabetes. In our sample, preferences for structured diabetes education and psychological support were linked with higher adherence scores, suggesting these patient-centered components may be particularly effective. Tailored education, brief psychological counselling, and shared decision-making should be incorporated into diabetes care to strengthen acceptance and support long-term self-management. Further studies are recommended to examine the effectiveness of acceptance-based interventions and to confirm these findings in larger and more diverse samples.

## Background

Diabetes is a chronic illness that significantly impacts both individual well-being and public health. It is often referred to as a civilization-related illness due to its growing prevalence worldwide. According to the International Diabetes Federation, approximately 537 million adults globally were diagnosed with diabetes in 2021 (1), with an increasing trend observed in many countries, including Poland. In 2023, an estimated 3 million Poles were affected by diabetes, underscoring the importance of effective prevention and management strategies (2).

The development of diabetes is affected by various genetic, environmental, and lifestyle factors. Major risk factors include an unhealthy diet, low physical activity, obesity, an aging population, stress, and environmental influences (3). The rising incidence of diabetes challenges healthcare systems, as it demands long-term medical treatment and lifestyle changes to prevent complications (2).

A crucial part of managing diabetes is the patient’s acceptance of their illness. Illness acceptance involves the mental and emotional process through which a person adapts to the restrictions caused by the disease, affecting their psychological health and how well they follow treatment (4). Greater levels of illness acceptance are linked to better self-care, increased adherence to medical advice, and overall treatment success. Patients who accept their condition more are more likely to actively participate in their therapy, resulting in better disease outcomes (4–6).

Another key factor in diabetes management is medication adherence, which refers to the extent to which patients follow prescribed treatment regimens. While adherence is often used interchangeably with compliance, these terms have distinct meanings. Adherence emphasizes the patient’s active participation in their treatment plan, whereas compliance suggests passive obedience to medical instructions. In the literature, adherence is generally preferred, as it reflects a collaborative approach to disease management (6).

Illness acceptance plays a crucial role in how patients manage chronic conditions such as diabetes. Higher levels of acceptance have been associated with better adherence to treatment regimens, including medication use and lifestyle modifications. Illness acceptance is considered a constructive psychological process that enables patients to adjust to their condition and actively participate in its management.

Coordinated care understood as coherent, team-based patient management by an interdisciplinary workforce (physician, nurse, diabetes educator, pharmacist, and psychologist) integrates the principles of holistic care, which addresses the patient’s biological, psychological, and social needs. Within this framework, self-management, i.e., the patient’s capacity to live with and manage the disease, is of central importance. However, observed shifts in the epidemiology of diabetes, changes in individual patient profiles, and rapid technological advances necessitate a transition toward a more individualized model of patient collaboration. This approach aligns with contemporary strategies for combating diabetes and can be described as a coordinated care model tailored to the individual with diabetes (7).

Given the significant role of both illness acceptance and medication adherence in diabetes management, this study aims to evaluate their levels among patients with diabetes and to identify factors influencing these aspects. Understanding the relationship between acceptance and adherence can inform healthcare professionals and policymakers about the need for targeted interventions, including psychological support and educational programs to enhance diabetes care. In this study, we posit that higher illness acceptance facilitates the implementation of treatment recommendations (adherence) by enhancing motivation, emotion regulation, and collaboration with the multidisciplinary care team, as well as engagement in one’s treatment. These studies concern research on a group of patients with diabetes and align with the concept of patient collaboration, referred to as preparing the patient for self-care.

## Materials and methods

### Subjects

The study involved 190 individuals, including 101 female and 89 male patients with type 1 and type 2 diabetes. Patients were approached consecutively during their scheduled outpatient clinic visits. Of 256 patients invited, 36 declined. Of the 220 questionnaires returned, 190 were fully completed and included in the final analysis (completion rate: 86%; usable rate: 74%). Data were collected from May 16, 2017 to December 31, 2017.

The study was conducted among patients of the Multispecialty Healthcare Center “GRYF-MED” in Bydgoszcz and the Endocrinology and Diabetology Outpatient Clinic of the Dr. Antoni Jurasz University Hospital No. 1 in Bydgoszcz. Participation was anonymous and voluntary.

Inclusion criteria: age ≥18 years; clinical diagnosis of diabetes (type 1 or type 2); minimum diabetes duration of at least 6 months since diagnosis; current use of at least one prescribed antidiabetic medication; ability to provide informed consent.

The study utilized an original questionnaire of its own design, consisting of 24 questions regarding sociodemographic factors, medical conditions, lifestyle, and treatment methods. Patients self-assessed their health status on a scale ranging from very good to very poor.

Standardized research tools were used in the study:

- The Acceptance of Illness Scale (AIS) questionnaire by Felton et al.—Polish adaptation by Juczyński (8)—included 8 statements accompanied by a 5-point response scale: 1—I strongly agree, 2—I agree, 3—I do not know, 4—I disagree, 5—I strongly disagree. The measure of the degree of acceptance of the current health status was the sum of all points, ranging from 8 to 40 points. The scores were divided into three ranges to determine the level of acceptance, grouped as follows: 8–18—no acceptance, 19–29—moderate acceptance, and 30–40—good acceptance of the illness.
- The eight-item Morisky Medication Adherence Scale (MMAS-8) was used as a diagnostic instrument to assess the level of adherence and compliance with medical recommendations—Polish adaptation by Jankowska-Polańska et al. (9). Respondents were asked to address 8 statements regarding them medication-taking routine. The total result obtained for all statements allowed us to assess the degree of adherence to the recommended drug regimen, where a score of <6 points indicated a low level, a score of ≥6 and <8 points indicated a moderate level, and 8 points indicated a high level of adherence and compliance (9–11).

### Study protocol

The study was conducted in accordance with the guidelines of the Declaration of Helsinki. The protocol received approval from the Bioethics Committee at Nicolaus Copernicus University Collegium Medicum in Bydgoszcz, Poland (consent number KB 362/2017). Each participant provided informed consent to take part in the study.

The consent for publication section is not applicable.

### Statistical analysis

Statistical analysis was performed using the Statistica v. 13 software (StatSoft, TULSA, USA). Basic qualitative (nominal) data were presented as the population size (*n*) and percentage (%). Non-parametric tests were applied for statistical analysis. The Spearman rank correlation test was used to assess correlations. The Mann–Whitney U test was used for comparisons between two groups, and the Kruskal–Wallis test was applied for comparisons among three or more groups. Quantitative (measurable) variables were presented as mean and standard deviation (M ± SD).

A total of 256 patients were invited to participate in the study, and 36 declined. Out of the 220 questionnaires returned, 190 were fully completed and were included in the final statistical analysis.

## Results

The general characteristics of the study population are presented in Table 1. The study involved 190 individuals, including 101 female and 89 male patients. The mean age of the participants was 42.2 ± 13.4 years: 41.3 years ± 13.9 for female patients, and 43.2 years ± 12.9 for male patients. The largest group of respondents had a secondary education (36.8%), while the smallest group had a primary education (9.5%). Among the respondents, 63.7% were employed. One-fifth of the patients (20.5%) lived alone. Among the study participants, 72.6% had type 2 diabetes. The mean time since the diagnosis of diabetes was 7.7 ± 5.7 years. In the study population, 87 patients (45.8%) declared the presence of coexisting diseases. When answering the question about the type of coexisting diseases, respondents were allowed to select more than one statement. Thus, a total of 122 responses were registered from 87 individuals. The most commonly reported coexisting conditions were hypertension (36.9%), followed by asthma (13.9%), obesity (8.2%), hypothyroidism (5.7%), and rheumatism (4.1%).

**Table 1:** The general characteristics of the study population.

Demographic characteristics of respondents	Group size *n* = 190 (%)
Gender	
Male	101 (53.2%)
Female	89 (46.8%)
Age (in years)	
Up to 30	49 (25.8%)
31–40	36 (18.9%)
41–50	44 (23.2%)
Over 50	61 (32.1%)
Place of residence	
Country	56 (29.5%)
City	134 (70.5%)
Self-assessment of health condition	
Very good	70 (36.9%)
Good	73 (38.4%)
Medium	42 (22.1%)
Bad	5 (2.6%)
Very bad	0 (0%)
Education	
Primary	18 (9.5%)
Vocational	59 (31.1%)
Secondary	70 (36.8%)
Higher	43 (22.6%)
Business activity	
Unemployment	24 (12.6%)
Full-time job	121 (63.7%)
Disability pension/retirement	27 (14.2%)
Pupil/student	18 (9.5%)
Living	
With family	151 (79.5%)
Alone	39 (20.5%)
Co-existing diseases	
Hypertension	45
Asthma	17
Obesity	10
Hypothyroidism	7
Rheumatism	5
Type of diabetes	
Type 1	52 (27.4%)
Type 2	138 (72.6%)

The average illness acceptance score among the patients was 29.16 ± 6.53 points. The study population was divided into three groups based on the degree of acceptance of their illness. The largest group consisted of individuals with a high level of illness acceptance—101 individuals (53.2%). Among the respondents, moderate acceptance of illness was reported by 75 individuals (39.5%). Fourteen individuals (7.4%) did not accept their illness.

Table 2 presents the mean values for individual statements on the Acceptance of Illness Scale, ranging from 3.34 to 4.07 points. The lowest average, and thus the lowest level of illness acceptance, was obtained for the statements: “My health condition makes me feel incomplete as a person” (3.34 points) and “I am having trouble adapting to the restrictions imposed by the disease” (3.35 points). The highest average, and consequently the highest level of acceptance of illness, was scored for the statements: “The disease makes me a burden to my family and friends” (4.07 points) and “Health problems make me more dependent on others than I would like to be” (4.06 points).

**Table 2:** Scores for individual Acceptance of Illness Scale statements.

No.	Statement	Mean	Standard deviation	Confidence −95.0%	Confidence +95.0%	Median
1	I am having trouble adapting to the restrictions imposed by the disease	3.35	1.134	3.19	3.51	3.0
2	Because of my condition, I cannot do what I like most	3.61	1.021	3.46	3.76	4.0
3	The disease sometimes makes me feel unnecessary	3.51	1.126	3.34	3.67	4.0
4	Health problems make me more dependent on others than I would like to be	4.06	0.944	3.92	4.19	4.0
5	The disease makes me a burden to my family and friends	4.07	0.965	3.93	4.21	4.0
6	My health condition makes me feel incomplete as a person	3.34	1.245	3.16	3.52	3.0
7	I will never be self-sufficient in the way I would like to be	3.84	1.059	3.69	3.99	4.0
8	I think the people around me often feel awkward about my illness	3.39	1.312	3.20	3.58	4.0

A detailed set of results on the levels of acceptance of illness in the study population is presented in Table 3. The highest average score for acceptance of illness was observed in the age group of 41–50 years, 29.75 points, and in those above 50 years of age, 29.56 points. The lowest score was observed in individuals aged up to 30 years, 28.43 points. Participants’ age, gender, education level, and type of diabetes did not show statistically significant correlations with the results of illness acceptance (*p* > 0.05).

**Table 3:** Detailed results on acceptance of illness in the study population.

Variable		Level of acceptance			*p*
		No acceptance	Moderate acceptance	High acceptance	
		Number (%)	Number (%)	Number (%)	
Age (in years)	Up to 30	2 (4.1%)	25 (51%)	22 (44.9%)	0.381
	31–40	4 (11.1%)	12 (33.3%)	20 (55.6%)	
	41–50	4 (9.1%)	15 (34.1%)	25 (56.8%)	
	Over 50	4 (6.6%)	23 (37.7%)	34 (55.7%)	
Gender	Female	4 (4%)	38 (37.6%)	59 (58.4%)	0.101
	Male	10 (11.2%)	37 (41.6%)	42 (47.2%)	
Level of education	Primary	2 (11.1%)	10 (55.6%)	6 (33.3%)	0.080
	Vocational	8 (13.6%)	20 (33.9%)	31 (52.5%)	
	Secondary	2 (2.9%)	29 (41.4%)	39 (55.7%)	
	Higher	2 (4.7%)	16 (37.2%)	25 (58.1%)	
Self-assessment of health condition	Very good	0 (0%)	26 (37.1%)	44 (62.9%)	0.003
	Good	8 (11%)	26 (35.6%)	39 (53.4%)	
	Medium and bad	6 (12.8%)	23 (48.9%)	18 (38.3%)	
Duration of diabetes (in years)	Up to 3	0 (0%)	11 (35.5%)	20 (64.5%)	0.020
	3–5	5 (10%)	14 (28%)	31 (62%)	
	6–10	2 (3.4%)	30 (50.8%)	27 (45.8%)	
	Over 10	7 (14%)	20 (40%)	23 (46%)	
Type of diabetes	Type 1	3 (5.8%)	28 (53.8%)	21 (40.4%)	0.106
	Type 2	11 (8%)	47 (34%)	80 (58%)	
Level of adherence and compliance	Low	7 (50%)	6 (42.9%)	1 (7.1%)	0.00001
	Moderate	27 (36%)	30 (40%)	18 (24%)	
	High	19 (18.8%)	37 (36.6)	45 (44.6%)	

When reporting the diabetes duration correlation (*R* = −0.168; *p* = 0.020), acknowledge this represents a small effect size. Statistical analysis revealed that the level of illness acceptance increased along with the self-assessment of health status (*R* = 0.35; *p* < 0.001). The highest average score for acceptance of illness was noted in individuals who rated their health as very good, 30.64 points, while the lowest was in the group who rated their health status as moderate, 27.28 points. In the study, the duration of diabetes was negatively correlated with illness acceptance (*r* = −0.168; *p* = 0.020), representing a small effect size. The highest average acceptance was observed among individuals with diabetes duration ≤3 years (30.94 points) and 3–5 years (29.7 points), and the lowest among those with duration >10 years (28.0 points). Therapeutic adherence and compliance in the study population were assessed using the MMAS-8 (Polish version). The results of illness acceptance showed a statistically significant positive correlation with the level of adherence and compliance (*R* = 0.29; *p* < 0.001), indicating a medium effect size. Consistent with this association, Table 3 shows a clear gradient: the highest mean adherence and compliance occurred in the high-acceptance group (6.88 points), whereas the lowest was observed in the low-acceptance group (5.23 points), with the medium group falling in between.

Answers to individual questions of the Morisky Medication Adherence Scale questionnaire are presented in Table 4. The mean total score was 6.49 ± 1.5, indicating a moderate level of adherence and compliance among the patients. Moderate level of adherence means that the patient generally follows medical recommendations regarding medication use, but does not always do so consistently. This level of adherence does not imply a lack of cooperation with the physician, but it may reduce the effectiveness of therapy, leading to fluctuations in blood glucose levels, poorer diabetes control, and an increased risk of complications.

**Table 4:** Answers to individual questions of the Morisky Medication Adherence Scale questionnaire.

Question	Mean	Standard deviation	Confidence −95.0%	Confidence +95.0%	Median
Do you sometimes forget to take your medications?	0.69	0.462	0.63	0.76	1.00
Sometimes people miss taking their medications for reasons other than forgetting. Over the past 2 weeks, were there any days when you did not take your medication?	0.92	0.270	0.88	0.96	1.00
Have you ever cut back or stopped taking your medications without telling your doctor because you felt worse when taking it?	0.79	0.405	0.74	0.85	1.00
When you travel or leave home, do you sometimes forget to take your medication with you?	0.71	0.457	0.64	0.77	1.00
Did you take all your medications yesterday?	0.94	0.244	0.90	0.97	1.00
When you feel like your symptoms are under control, do you sometimes stop taking your medication?	0.80	0.401	0.74	0.86	1.00
Taking medication every day is a real inconvenience for some people. Do you ever feel hassled about sticking to your treatment plan?	0.75	0.436	0.69	0.81	1.00
How often do you have difficulty remembering to take all your medications?	0.89	0.175	0.87	0.92	1.00

Among all items assessing medication adherence, the highest average score was given to question 5: “Did you take all your medications yesterday?” (0.94 points). This was followed closely by question 2: “Sometimes people miss taking their medications for reasons other than forgetting. Over the past 2 weeks, were there any days when you did not take your medication?” (0.92 points) (Table 4 for detailed responses).

Table 5 presents the detailed results of therapeutic adherence and compliance assessed using the Morisky Medication Adherence Scale. Statistical analysis showed that with increasing age of the participants, the level of adherence and compliance decreased (*R* = −0.17; *p* = 0.016). A higher mean score for adherence and compliance was observed in the female group, as compared to males (6.85 ± 1.36 vs. 6.08 ± 1.59 points, *p* < 0.001). With higher levels of education of the participants and self-assessment of their health status, the level of adherence and compliance also increased (*R* = 0.29; *p* < 0.001 for both). The duration of diabetes treatment did not affect the level of adherence and compliance (*p* > 0.05). A higher mean score for adherence and compliance was observed in individuals with type 1 diabetes, as compared to those with type 2 (6.99 vs. 6.31 points, *p* = 0.003). The level of acceptance of illness influenced the level of adherence and compliance (*R* = 0.29; *p* < 0.0001).

**Table 5:** Detailed results on therapeutic adherence and compliance in the study population.

Variable		Level of adherence and compliance			*p*
		Low	Moderate	High	
		Number (%)	Number (%)	Number (%)	
Age (in years)	Up to 30	9 (18.4%)	18 (36.7%)	22 (44.9%)	0.016
	31–40	11 (30.6%)	9 (25%)	16 (44.4%)	
	41–50	15 (34.1%)	17 (38.6%)	12 (27.3%)	
	Over 50	18 (29.5%)	29 (47.5%)	14 (23%)	
Gender	Female	22 (21.8%)	30 (29.7%)	49 (48.5%)	<0.001
	Male	31 (34.8%)	43 (48.3%)	15 (16.9%)	
Level of education	Primary	12 (66.7%)	4 (22.2%)	2 (11.1%)	<0.001
	Vocational	17 (28.8%)	28 (47.5%)	14 (23.7%)	
	Secondary	18 (25.7%)	25 (35.7%)	27 (38.6%)	
	Higher	6 (14%)	16 (37.2%)	21 (48.8%)	
Self-assessment of health condition	Very good	10 (14.3%)	26 (37.1%)	34 (48.6%)	0.001
	Good	25 (34.2%)	32 (43.8%)	16 (21.9%)	
	Medium and bad	18 (38.3%)	15 (31.0%)	14 (29.8%)	
Duration of diabetes (in years)	Up to 3	8 (25.8%)	15 (48.4%)	8 (25.8%)	0.81
	3–5	13 (26%)	20 (40%)	17 (34%)	
	6–10	17 (28.8%)	21 (35.6%)	21 (35.6%)	
	Over 10	15 (30%)	17 (34%)	18 (36%)	
Type of diabetes	Type 1	10 (19.2%)	15 (28.8%)	27 (51.9%)	0.003
	Type 2	43 (31.2%)	58 (42%)	37 (26.8%)	
Acceptance of Illness Scale score	No acceptance	7 (50%)	6 (42.9%)	1 (7.1%)	0.00001
	Moderate	27 (36%)	30 (40%)	18 (24%)	
	Acceptance				
	High acceptance	19 (18.8%)	37 (36.6)	45 (44.6%)	

For a patient with diabetes, it is important to monitor and control their glucose levels at home. Among those surveyed, the vast majority (76.8%) declared that they performed such monitoring. Patients most commonly measured their glucose levels once a day (33.6%), then three times a day (26.7%), and lastly twice a day (26.0%). As many as 65.8% of the respondents reported receiving assistance from family members in glucose monitoring. Monitoring glucose and blood pressure is associated with keeping a self-monitoring diary where these values are recorded. Keeping such a diary was declared by 64.7% of the patients. Among 76 patients treated with subcutaneous insulin, 76.3% indicated self-preparation and administration of injections. All patients receiving insulin through an insulin pump (39 individuals) were able to independently operate the equipment thanks to the training they had received.

## Discussion

Diabetes is a chronic condition in which treatment effectiveness largely depends on active patient participation. In such a model of care, adherence and compliance are pivotal, as they determine the maintenance of recommendations in everyday practice.

The primary aim of this study was to evaluate the level of illness acceptance and adherence to therapeutic recommendations among patients with diabetes and to identify the factors influencing these aspects. Our findings demonstrate a significant positive correlation between illness acceptance and medication adherence (*R* = 0.29; *p* < 0.001), supporting previous research (12, 13). Patients with higher illness acceptance scores (mean: 6.88 points) exhibited better adherence compared to those with low acceptance (5.23 points). These findings highlight the crucial role of psychological adaptation in managing diabetes.

Cognitive function -are an important determinant of adherence to therapeutic recommendations, although cognitive decline likely did not play a major role in our relatively young study sample (14, 15). Deficits in memory, executive functions, and health literacy (reading and writing skills) are associated with poorer adherence (16–18). Patients with type 1 diabetes demonstrated higher mean adherence scores than those with type 2 diabetes (6.99 ± 1.36 vs. 6.31 ± 1.59 points, *p* = 0.003). This aligns with earlier findings that dependence on insulin for survival promotes greater adherence (19). At the same time, psychosocial aspects—including health beliefs, perceived severity, and self-efficacy—play a crucial role in adherence differences (20–25). According to the Health Belief Model, patients are more likely to comply when they perceive a high risk of complications and believe in treatment efficacy (23), while psychological insulin resistance (PIR) may emerge when insulin initiation is perceived as a failure, reducing adherence (26, 27).

Our study found a statistically significant negative correlation between diabetes duration and illness acceptance (*R* = −0.168; *p* = 0.020), indicating that patients with a longer disease duration had lower acceptance levels. A study of 298 Turkish patients reported a similar lack of correlation between age and illness acceptance, but higher acceptance among women, more educated patients, and those without comorbidities (12). Khazew and Faraj (28) also found no consistent sociodemographic predictors, suggesting that cultural or contextual factors may influence illness perception. Future studies should include larger and more diverse populations to explore these associations further.

Socioeconomic factors such as education, healthcare access, and financial barriers also shape adherence (29, 30). Our results confirmed the positive association between higher education and adherence (*R* = 0.29; *p* < 0.001), consistent with reports that health literacy improves treatment engagement (31). Patients with type 1 diabetes often benefit from more structured education and early training, while those with type 2 may lack adequate support, increasing the risk of non-adherence (31). Addressing these disparities through targeted education and support programs may help bridge this gap.

Future research should investigate psychosocial determinants such as health beliefs, self-efficacy, and barriers to adherence, and assess the effectiveness of tailored interventions, including cognitive-behavioral, motivational, educational, and acceptance-based approaches, in larger and more diverse populations (32). Given the rising incidence of both type 1 and type 2 diabetes, a coordinated care model is being implemented in response to system-level needs. This model directs resources toward individualized education, continuous follow-up, and psychosocial support, aligning with the patient needs identified in our findings.

Coordinated diabetes care may also enhance both acceptance and adherence. In Poland, the coordinated care model introduced in 2023 integrates physicians and nurses in continuous patient-centered care, aligning with current clinical recommendations of the Polish Diabetes Association.

## Conclusion

In the present study, it was found that patients with diabetes exhibited a moderate level of adherence and compliance and a high level of acceptance of illness. Patients who accepted their diabetes tended to demonstrate better self-care and adherence to medical recommendations. Higher education of patients correlated with a greater level of acceptance of illness and better adherence to medical recommendations. Patients with type 1 diabetes showed a higher level of adherence to recommendations than those with type 2 diabetes, which may result from intensive education on insulin therapy.

Monitoring disease acceptance and adherence to treatment recommendations can be an important element of diabetes care. Periodic monitoring, especially during health and family crisis situations, would be beneficial. Coordinated diabetes care aims to minimize or delay complications, maintain a good quality of life for the patient, and reduce healthcare costs for both the patient and the healthcare system.

### Practice implications

Identification of patients with diabetes who experience difficulties adhering to therapeutic recommendations allow for the development of individual treatment plans and the provision of additional education and support, and also reduces the risk of diabetes complications. Based on the findings of this study, strategies for diabetes patient care can be tailored to focus on promoting acceptance of illness and supporting self-management. Educational programs and psychosocial support initiatives can be implemented to enhance diabetes management among patients. The study results can be valuable for healthcare policymakers and administrators involved in planning the provision of healthcare services to patients with diabetes, as they indicate the need to increase access to educational programs and psychological support for these patients.

In the Polish context, the Self-Care of Diabetes Inventory (SCODI)—in its Polish adaptation—can be used to assess areas of self-care, alongside the MMAS-8 (medication adherence) and AIS (illness acceptance).

### Limitations

The authors were unable to utilize glycemia and glycosylated hemoglobin values in the study because patients used various equipment for self-monitoring of glycemia. Authors were also unable to perform glycemia and glycosylated hemoglobin assays because patients attended medical appointments at different times of the day (patients were not fasted). Therefore, such test results would not be reliable.

Given the cross-sectional design, we cannot draw causal conclusions about the relationship between illness acceptance and adherence, nor determine its directionality. We did not assess the presence or severity of diabetes complications, which could influence both illness acceptance and adherence; thus, we could not adjust for potential confounding by disease burden.

## Data Availability

The raw data supporting the conclusions of this article will be made available by the authors, without undue reservation.

## Author’s note

The results of this study were presented at the International Conference on Research in Life-Sciences & Healthcare, Paris, France, 23-24 April 2021.
